# Toxicological Effects of Combined Exposure of Cadmium and Enrofloxacin on Zebrafish

**DOI:** 10.3390/toxics13050378

**Published:** 2025-05-07

**Authors:** Lingfei Ren, Yu He, Chao Hou, Chaoxuan Liao, Miao Chen

**Affiliations:** 1 College of Resources and Environmental Engineering, Guizhou University, Guiyang 550025, China; gs.lfren22@gzu.edu.cn; 2 Guizhou Academy of Testing and Analysis, Guiyang 550014, China; 3 College of Ecological Environment Engineering, Guizhou University for Nationalities, Guiyang 550025, China; 4 Guizhou Karst Environmental Ecosystems Observation and Research Station, Ministry of Education, Guiyang 550025, China; 5 Key Laboratory of Karst Georesources and Environment, Ministry of Education, Guiyang 550025, China

**Keywords:** antibiotics, heavy metals, combined impact, antioxidant enzymes, intestinal flora

## Abstract

The combined pollution of cadmium (Cd) and enrofloxacin (ENR) in aquatic environments represents a critical issue in environmental toxicology. Using zebrafish as model organisms, we systematically investigated the combined toxicity of Cd and ENR through both acute (96-h) and chronic (20-d) exposure experiments. Our results demonstrated significant synergistic effects: co-exposure reduced the 96-h LC50 values from 89.12 mg/L (Cd alone) and 190.11 mg/L (ENR alone) to 46.35 mg/L and 99.39 mg/L, respectively (combined effect index = 0.96). Chronic exposure revealed that ENR enhanced Cd accumulation in the liver, intestine, and muscle tissues by 1.11–2.33-fold compared to single Cd exposure. Oxidative stress markers showed dynamic temporal changes, with superoxide dismutase (SOD), glutathione peroxidase (GSH-Px), and catalase (CAT) activities initially increasing by 1.34–7.06-fold, 0.98–3.28-fold, and 1.53–3.65-fold at 8 d, respectively, followed by 9.9–48.98% reductions after 20 d of exposure. Malondialdehyde (MDA) levels progressively accumulated, reaching up to 4.06-fold higher than controls. Notably, co-exposure elevated oxidative stress by 11.24–34.48% relative to single exposures. The 16S rDNA sequencing analysis indicated that Cd exposure significantly reduced the α-diversity of zebrafish gut microbiota (57–63% decrease in Shannon index), characterized by a 16–20% reduction in beneficial *Cetobacterium* and a 44–114% increase in pathogenic *Aeromonas* abundance. The combined exposure further exacerbated these gut microbiota dysbiosis patterns. These findings provide crucial evidence for ecological risk assessment, suggesting that current environmental standards based on single-pollutant evaluations may substantially underestimate the actual risks of heavy metal-antibiotic co-contamination in aquatic ecosystems.

## 1. Introduction

Currently, antibiotics have been widely used in the medical, aquaculture, and livestock industries as inhibitors of pathogenic microorganisms or growth promoters [1]. Enrofloxacin (ENR) is a fluoroquinolone antibiotic with the molecular formula C_19_H_22_FN_3_O_3_. It exerts its antibacterial effects by inhibiting the activity of bacterial DNA gyrase and topoisomerase IV, thereby disrupting the DNA replication process in bacteria [2]. Due to its broad-spectrum antimicrobial activity, ENR is broadly applied in the prevention and treatment of various animals’ infectious diseases. After administration to animals, ENR is often not completely absorbed, with the majority being excreted in urine and feces [3]. These excretions then enter aquatic environments through soil infiltration, surface water runoff, atmospheric deposition, rain wash, and other pathways, causing water pollution. ENR has been widely detected in surface water, groundwater, and drinking water, with concentrations ranging from 0.019 to 0.229 μg/L in the waters of Gonghu Bay, Taihu Lake, China [4], and from 2.0 to 4.0 ng/L in drinking water in Guangzhou and Macao [5], ENR was detected in rivers and farm wastewater across six provinces in Argentina, with concentrations ranging from 0.97 μg/L to 11.9 μg/L [6]. The presence of ENR in water bodies not only induces bacterial resistance but also poses potential toxicity to other organisms [7]. The widespread use of ENR not only exerts negative impacts on aquatic ecosystems, such as promoting the spread of antibiotic resistance [8], inducing toxicity to aquatic organisms [9], and disrupting biological chains [10] but may also affect human public health through the water cycle [11].

Heavy metals have become widely considered pollutants due to their toxicity, persistence, and bioaccumulation [12,13]. Cadmium (Cd) is widely used in manufacturing, mining, and agriculture because of its excellent corrosion resistance and chemical stability [14,15]. With the acceleration of industrialization, Cd concentrations in water bodies have rapidly increased; studies have shown that elevated concentrations of Cd have been detected in water bodies and surface sediments of industrial coastal areas. For instance, in the coastal region of Zhejiang, China, Cd concentrations reached 4.78 μg/L in water and 0.43 mg/kg in surface sediments [16], while in the Arabian Gulf region of Saudi Arabia, Cd concentrations in sediments were as high as 4.48 mg/kg [17]. Although China’s national quality standard for surface water, “Environmental Quality Standards for Surface Water” (GB 3838-2002) [18], stipulates that Cd content should not exceed 10 μg/L in Class V surface water (designated for agricultural and general landscape use) and 5 μg/L in water used for agriculture and fisheries, actual Cd concentrations in water bodies are often much higher than these national standards [19], inevitably threatening ecological balance and aquatic organism health. Studies have shown that even at extremely low concentrations in aquatic environments (According to the relevant regulations of the European Union, the maximum allowable concentration of cadmium in freshwater environments is 0.25 µg/L [refer to EU Directive 2013/39/EU]. Additionally, Lee et al. [20] have indicated that ENR concentrations as low as 5 µg/L may pose ecological risks to aquatic organisms.), Cd can be toxic to algae [21], benthic organisms [22], and fish [23], and may even pose potential threats to humans through the food chain [24].

Numerous studies have shown that exposure to ENR and Cd alone can affect the physiological performance of aquatic organisms such as fish and shrimp to varying degrees, such as disrupting antioxidant systems [25,26], altering intestinal flora [9,27], accumulating in organisms [28,29], and affecting gene expression [30,31]. Studies by Alvarado [32] and Chen [33] have demonstrated that Cd and ENR can accumulate in the liver, kidneys, gills, and muscles of carp. Research by Wang et al. [27] has shown that exposure to Cd significantly alters the composition of intestinal microbiota in crucian carp, while Chen et al. [34] found that exposure to ENR markedly reduces the diversity of intestinal microbial communities in tilapia, leading to a decrease in dominant bacterial populations within the gut microbiota. Additionally, some studies have reported the combined effects of antibiotics and heavy metals on soil organisms [35,36,37]. Research indicates that the complexation of heavy metals and antibiotics can significantly alter the absorption of antibiotics or heavy metals [38,39]. However, there are few reports on the toxic effects of combined exposure to ENR and Cd on aquatic organisms, and it remains unclear whether there is an interaction between them. Zebrafish (*Danio rerio*), as a typical aquatic model organism, shares a high degree of homology with human genes and is sensitive to exogenous compounds. The toxicity and teratogenic effects of pollutants on zebrafish are similar to those on humans in many ways, making them widely used in environmental toxicology research [40]. In this study, zebrafish were used as the research subject to explore the combined toxicity of ENR and Cd by examining the dynamic changes in Cd content in the liver, intestine, and muscle tissues of zebrafish, the activities/contents of enzymes related to oxidative stress, and changes in intestinal flora. The aim is to provide a reference for assessing the ecological risk of combined pollution by ENR and Cd to aquatic organisms.

## 2. Materials and Methods

### 2.1. Chemical Reagents

Enrofloxacin (AR) was purchased from Shanghai Macklin Biochemical Co., Ltd. (Shanghai, China), cadmium chloride (AR) was obtained from Tianjin Komiou Chemical Reagent Co., Ltd. (Tianjin, China), and nitric acid and hydrogen peroxide (GR) were acquired from Sinopharm Chemical Reagent Co., Ltd. (Shanghai, China). Superoxide dismutase (SOD), catalase (CAT), glutathione peroxidase (GPx), malondialdehyde (MDA), and total protein (TP) assay kits were all purchased from Nanjing Jiancheng Bioengineering Institute (Nanjing, China).

### 2.2. Experimental Process

A total of 6000 zebrafish (AB strain) aged 3 to 4 months were purchased from Shanghai Hongye Ornamental Fish Farm and randomly distributed into ten 200 L tanks. The fish were acclimatized for 15 days under experimental conditions. During the acclimatization period, the fish were fed twice daily with a feed amount equivalent to 1% of their body weight, and residual feed was promptly removed. The rearing water was aerated for 48 h, filtered using quartz balls, and sterilized with ultraviolet light. The water temperature was maintained at 25 ± 1 °C, with pH stabilized at 7.2–7.5, and a light-dark cycle of 14 h light: 10 h dark. One-third of the rearing water was replaced daily, and a complete water change was performed weekly to maintain water quality stability and prevent disease outbreaks in zebrafish. This experiment was conducted in strict accordance with the protocol approved by the Laboratory Animal Management Committee of the Guizhou Provincial Analytical Testing Research Institute (Approval No. BMY-ZY-202402).

The experiment was conducted according to the static test method specified in the “Chemicals—Acute Toxicity Test for Fish” (GB/T 27861-2011) [41]. Preliminary experiments were performed to determine the maximum non-lethal concentration and the minimum lethal concentration of Cd and ENR for zebrafish over a 96-h period. Based on these results, six gradient concentrations were established between these two thresholds using equal intervals. Cd exposure concentrations: 31, 49, 68, 86, 104, and 122 mg/L; ENR exposure concentrations: 120, 160, 240, 280, and 320 mg/L. The acute toxicity test was carried out in 8 L glass tanks, with an effective liquid volume of 4 L per tank. Each concentration was tested in triplicate, with 10 zebrafish per replicate, totaling 360 fish. Additionally, a blank control group was included. Mortality was recorded at 24 h, 48 h, 72 h, and 96 h. The criteria for determining zebrafish death were as follows: the fish exhibited a supine or sideways floating posture on the water surface, lost the ability to swim actively, and showed no response to needle stimulation. Feeding was suspended 24 h prior to the experiment, and no feeding was provided during the acute toxicity test. Mortality rates were calculated based on the number of deaths observed at the end of the experiment.

Based on the 96-h median lethal concentration (LC_50_) of Cd and ENR under individual exposure, the exposure concentration gradients for Cd and ENR were established using a geometric ratio of 1.5. The combined exposure solutions were prepared at a Cd:ENR ratio of 1:2.1, with the following concentration pairs: 18 mg/L Cd + 37 mg/L ENR, 27 mg/L Cd + 56 mg/L ENR, 40 mg/L Cd + 84 mg/L ENR, 60 mg/L Cd + 126 mg/L ENR, 90 mg/L Cd + 190 mg/L ENR, 135 mg/L Cd + 285 mg/L ENR. Each treatment group had 3 replicates, and the experimental procedure was the same as described above.

When conducting the chronic toxicity experiment, we referred to the 96 h-LC_50_ value of the combined exposure. Based on this value, we selected concentrations of 1/100 and 1/1000 of it, respectively, to set up four experimental groups. Meanwhile, a blank control group was established. These groups included the CK group (control group), L-Cd group (Cd 0.046 mg/L), H-Cd group (Cd 0.46 mg/L), L-CE group (Cd 0.046 mg/L and ENR 0.099 mg/L), and H-CE group (Cd 0.46 mg/L and ENR 0.99 mg/L). A total of 3900 zebrafish that completed acclimatization were randomly allocated to experimental tanks, with three replicates per group and 260 fish per tank. During the experiment, the fish were fed twice daily (at 10:00 AM and 6:00 PM) for a continuous exposure period of 20 days. Cd and ENR were added according to the designed experimental concentrations, and the rearing water was replaced every four days. To ensure the stability of the experimental concentrations, water samples were collected before and after each water change. The Cd concentration was measured using ICP-MS (Agilent 7800, Santa Clara, CA, USA), and the ENR concentration was detected using UHPLC-MS/MS (Agilent 1290-6470A, USA). The concentrations of Cd and ENR in the control group were below the detection limits of the instruments, while the concentrations in the experimental groups remained within 80% to 120% of the theoretical values. Samples were collected on days 0, 1, 2, 4, 6, 8, 12, 16, and 20 for the determination of Cd content in zebrafish; on days 1, 8, and 20 for the measurement of antioxidant enzymes in the liver; and on day 20 for the assessment of intestinal microbial diversity. Random sampling was conducted each time, and the collected fish samples were rinsed with ultrapure water, euthanized on ice, and dissected on ice-packed trays. The intestines, liver, and muscles were rapidly separated, weighed, and stored at −80 °C for subsequent use.

### 2.3. Analytical Method

#### 2.3.1. Joint Toxicity Assessment Method

The toxic interaction between Cd and ENR was assessed using the Concentration Addition (CA) model [42] and the deviation ratio of the Multiple Dose Response (MDR) model [43,44]. The calculation formulas are as follows:

(1) $$EC_{x,mix}={\left(\sum_{i=1}^{n}\frac{p_{i}}{EC_{x,i}}\right)}^{-1}$$

where *EC_x_*_,*mix*_ represents the concentration of the mixture that produces effect *x*; *ECx*,*i* represents the concentration of the ith compound in the mixture when it is present alone and produces effect *x*. *p_i_* is the proportion of the concentration of the ith component in the mixture to the total concentration of the mixture.

(2) $$MDR=\frac{EC_{x,PRE}}{EC_{x,OBS}}$$

(3) $${MDR}_{Upper}=\frac{EC_{x{,}_{Upper}}}{EC_{x,OBS}}$$

(4) $$MDR_{Lower}=\frac{EC_{x{,}_{Lower}}}{EC_{x,OBS}}$$

MDR stands for the Model Deviation Ratio, which quantifies the deviation between the model-predicted values and the experimentally observed values. *EC_x_*_,*PRE*_ is the model-predicted concentration of the mixture that produces effect *x*. *EC_x_*_,*OBS*_ is the actual concentration of the mixture that produces effect *x*. *EC_x_*_,*Upper*_ and *EC_x_*_,*Lower*_ are the upper and lower limits, respectively, of the 95% confidence interval for *EC_x_*_,*OBS*_. Under a specified effect, synergy is indicated when MDR is greater than the upper limit of MDR (*MDR_Upper_*), antagonism is indicated when MDR is less than the lower limit of MDR (*MDR_Lower_*), and addition is indicated when MDR falls between the upper and lower limits of MDR.

#### 2.3.2. Determination of Cd in Tissues (ng/L) and Organs of Zebrafish

The weighed liver, intestine, and muscle samples were placed in polytetrafluoroethylene digestion vessels. To each vessel, 8 mL of nitric acid and 2 mL of hydrogen peroxide were added, and the reaction was allowed to proceed statically for 4 h before being subjected to digestion in a microwave digestion instrument (Mars6, CEM Corporation, Matthews, NC, USA). Upon completion of the digestion, the liquid in the digestion vessels was transferred and diluted to a fixed volume in 50 mL centrifuge tubes, which were then stored in a refrigerator at 4 °C until analysis. The concentration of Cd in the samples was determined using an inductively coupled plasma mass spectrometer (Agilent 7800, USA).

#### 2.3.3. Determination of Antioxidant Stress Index

The liver was transferred into a centrifuge tube and homogenized using 0.86% cold saline as the homogenization medium at a ratio of tissue sample fresh weight (g) to homogenization medium volume (mL) of 1:9 to prepare a 10% tissue homogenate of the liver. The homogenate was centrifuged at high speed in a refrigerated centrifuge (Sorvall Legend Micro 21R, Thermo Fisher Scientific, Waltham, MA, USA) at 4 °C for 10 min, and the supernatant was collected for the determination of various indicators. The levels of superoxide dismutase (SOD), catalase (CAT), glutathione peroxidase (GSH-PX), malondialdehyde (MDA), and total protein (TP) were measured strictly in accordance with the instructions provided with the assay kits. All kits were purchased from the Nanjing Jiancheng Bioengineering Institute (China). The absorbance was measured using a full-wavelength scanning microplate reader (Multiskan GO, Thermo Fisher Scientific, USA), and the activities of the relevant enzymes were calculated according to the manufacturer’s instructions.

#### 2.3.4. Determination of 16S rRNA Gene of Intestinal Flora of Zebrafish

The V3–V4 region of zebrafish gut microbiota 16S rRNA genes were amplified using specific primers 338F/806R (Sangon Biotech, Shanghai, China), with the amplification products subsequently verified by agarose gel electrophoresis and purified using a DNA purification kit. Following quantification with Qubit 4.0, sequencing libraries were constructed and subjected to high-throughput sequencing on the Illumina Nextseq2000 platform (Majorbio, Shanghai, China). The raw sequencing data underwent quality control processing through the Majorbio Cloud Platform (www.majorbio.com), where operational taxonomic units (OTUs) were clustered at 97% similarity using the UPARSE algorithm and taxonomically annotated against the SILVA database (v138). All statistical analyses were performed using the following R packages: “vegan” for α-diversity indices (Shannon, Simpson), β-diversity analysis (Bray-Curtis distance-based PCA/PCoA), and inter-group differential analysis of species abundance at phylum and genus levels; “LEfSe” for biomarker discovery (LDA score > 3.0, *p* < 0.05); and “Hmisc” combined with “igraph” for microbial co-occurrence network construction (Spearman’s correlation |r| > 0.6, *p* < 0.05). Network topological properties, including edge number, connectivity, node degree, and average clustering coefficient, were calculated to evaluate network complexity.

#### 2.3.5. Data Analysis

The experimental data were plotted using Origin 2020 software and the Majorbio platform. Prior to statistical analysis, all datasets were tested for normality (Shapiro-Wilk test, *p* > 0.05) and homoscedasticity (Levene’s test, *p* > 0.05). The 96-h LC_50_ values and their 95% confidence intervals were calculated through probit-concentration linear regression analysis using Fieller’s theorem in SPSS 2019. For normally distributed data, one-way ANOVA with Tukey’s post-hoc test was performed using SPSS 2019 (statistical significance at *p* < 0.05 was denoted by different letters and asterisks “*”). All data are presented as mean ± standard deviation (SD).

## 3. Results

### 3.1. Acute Toxicity of Cd and ENR to Zebrafish

**Figure 1:**
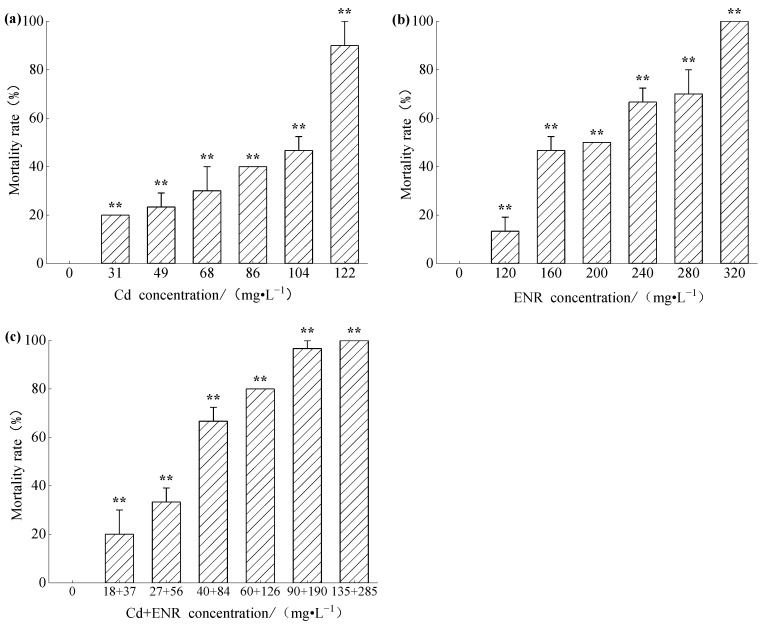
Mortality of zebrafish exposed to (**a**) Cd alone treatment group, (**b**) ENR alone treatment group, and (**c**) combined treatment group for 96 h (Note: “**” Indicates a statistically significant difference between the experimental group and the control group (*p* < 0.01), Due to the low variability among replicates in some experimental groups, error bars did not show significant differences and were therefore not included in the figures).

The mortality results of zebrafish exposed to different concentrations of Cd solution for 96 h are shown in Figure 1a. As can be seen from Figure 1, the mortality rates of zebrafish in all concentration treatment groups exhibited an upward trend. The calculated 96 h-LC_50_ for Cd to zebrafish was 89.12 mg/L. Compared with the control group, there were significant differences in mortality rates across all concentrations, with *p* < 0.01. The mortality results of zebrafish exposed to different concentrations of ENR solution for 96 h are depicted in Figure 1b. Figure 1b indicates a clear concentration-effect relationship between enrofloxacin concentration and zebrafish mortality. The calculated 96 h-LC_50_ value for ENR to zebrafish was 190.11 mg/L. Compared with a mortality rate of 0% in the control group, there were significant differences in mortality rates across all concentration groups.

Using the single toxicity of Cd and ENR to zebrafish as a reference, acute toxicity experiments were conducted with combined exposure to different concentrations of Cd and ENR. The mortality rates of zebrafish exposed to different combined concentrations for 96 h are shown in Figure 1c. The calculated 96-h LC_50_ values for Cd and ENR during co-exposure were determined to be 46.35 mg/L and 99.39 mg/L, respectively, for zebrafish. The toxicity of the combined exposure was 1.92 times that of Cd alone and 1.91 times that of ENR alone.

Based on the LC_50_ values for single exposure to Cd and ENR and their combined exposure, the predicted combined exposure effect concentration was calculated to be 139.91 mg/L using the Concentration Addition (CA) model. The Model Deviation Ratio (MDR) was 0.96, which falls within the confidence interval of MDR (0.58 to 1.04), indicating that the combined effect of Cd and ENR was additive.

### 3.2. Cd Accumulation in Zebrafish Tissues Under ENR Conditions

**Figure 2:**
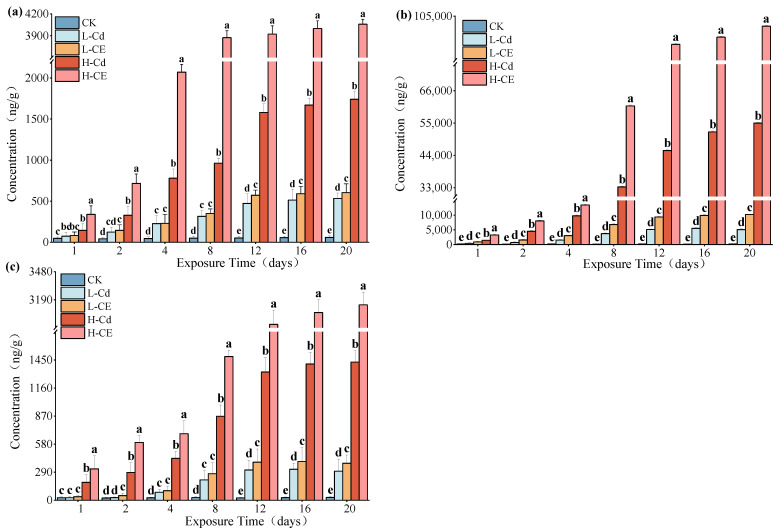
Effects of Cd single exposure and combined exposure to Cd and ENR on Cd content in (**a**) liver, (**b**) intestine and (**c**) muscle tissues of zebrafish (Note: Different lowercase letters refer to differences at significance level *p* < 0.05 among different exposure groups).

The concentrations of Cd in the liver, intestine, and muscle of zebrafish were determined, and the results are presented in Figure 2. As evident from Figure 2, when exposed for the same duration, the order of Cd content in different tissues of zebrafish is intestine > liver > muscle. Moreover, with prolonged exposure time and increased exposure concentration, the Cd content in various tissues of zebrafish showed an increasing trend. Within the same exposure duration, the Cd concentrations in all tissues of zebrafish in the combined exposure group were higher than those in the group treated with Cd alone. At the peak exposure time of 20 days, in the low-concentration groups (Cd 0.046 mg/L and Cd 0.046 mg/L + ENR 0.099 mg/L), the contents of Cd in the liver, intestine, and muscle of the combined exposure group increased by 1.13-, 1.20-, and 1.27-fold, respectively. In the high-concentration groups (Cd 0.46 mg/L and Cd 0.46 mg/L + ENR 0.99 mg/L), the contents of Cd in the corresponding tissues of the combined exposure group increased by 2.33-, 2.20-, and 2.26-fold, respectively. ENR exhibits a significant promoting effect on the absorption and accumulation of Cd in various tissues of zebrafish.

### 3.3. Effect of Cd on Oxidative Stress Indexes in Liver Under ENR

**Figure 3:**
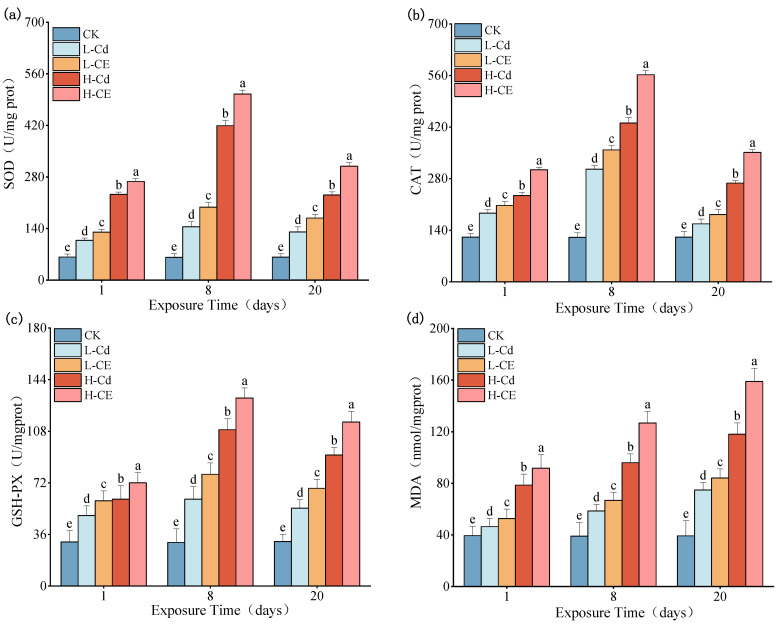
Effects of Cd single exposure and combined exposure to Cd and ENR on (**a**) SOD, (**b**) cat, (**c**) GSH-Px and (**d**) MDA activity/content in zebrafish liver (Note: Different lowercase letters refer to differences at significance level *p* < 0.05 among different exposure groups).

Effects of individual Cd exposure and combined Cd-ENR exposure on the antioxidant system and lipid peroxidation in zebrafish liver are presented in Figure 3. Compared to the control group, all exposure groups showed significantly elevated activities of SOD, CAT, and GSH-Px, along with increased MDA content (*p* < 0.05). Notably, the activities of these three antioxidant enzymes exhibited a biphasic response pattern (initial increase followed by decline) with prolonged exposure duration, while MDA content demonstrated a time-dependent accumulation pattern.

In the co-exposure groups, all measured parameters were significantly higher than those in the corresponding single cadmium exposure groups (*p* < 0.05). Specifically, in the low-concentration groups (L-Cd vs. L-CE), after 1, 8, and 20 days of exposure, SOD activity increased by 20.36%, 36.62%, and 28.82%, respectively; CAT activity showed increases ranging from 11.24% to 17.15%; GSH-Px activity was elevated by 20.78–28.53%; and MDA content rose by 12.37–14.40%. Similarly, in the high-concentration groups (H-Cd vs. H-CE), SOD activity increased by 15.12–33.80%, CAT activity by 29.87–31.06%, and GSH-Px activity by 18.80–25.11% at the corresponding time points. Notably, MDA content exhibited more pronounced increases of 16.66–34.45% in the high-concentration co-exposure groups.

### 3.4. Effect of Cd and ENR on Intestinal Flora Diversity

**Figure 4:**
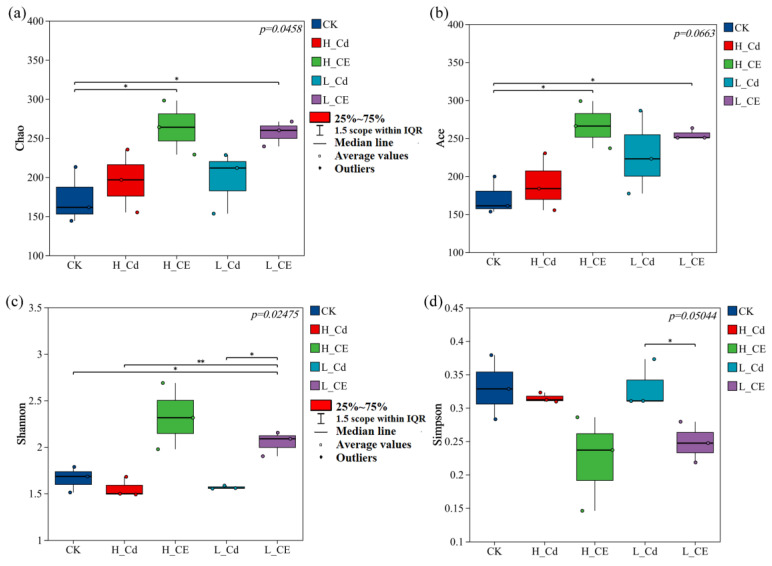
α-diversity indices of intestinal microbiota in zebrafish after 20 days of Cd single exposure and combined exposure to Cd and ENR. (**a**) Chao, (**b**) ACE, (**c**) Shannon, and (**d**) Simpson indices. (Note: The *p*-value < 0.05 in the upper right corner indicates that the index has significant differences between groups. “*” and “**” indicate significant and highly significant differences between groups, respectively).

**Figure 5:**
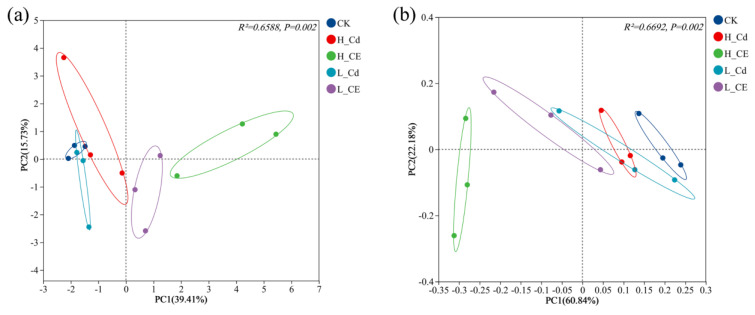
Beta diversity analysis of intestinal microbiota in zebrafish after 20 days of Cd single exposure and combined exposure to Cd and ENR. (**a**) PCA analysis, (**b**) PCoA analysis. (Note: Points of different colors represent samples of different groups. The closer the two sample points are, the more similar the species composition of the surface sample is).

The results of four α-diversity indices revealed the richness and diversity of the intestinal microbiota of zebrafish across different treatment groups. Specifically, the Chao index indicated that the H-CE treatment group harbored the highest number of intestinal bacteria in zebrafish, followed by the L-CE treatment group, then the L-Cd treatment group, with the H-Cd treatment group ranking second (Figure 4a). The ACE index demonstrated a significant difference in species richness between the CK group and the combined treatment groups (Figure 4b). Compared to the CK group, the Shannon index decreased in both the L-Cd and H-Cd treatment groups, while it increased in the combined treatment groups compared to the L-Cd and H-Cd groups (Figure 4c). When compared to the CK group, the Simpson index decreased in the single Cd treatment groups, whereas it decreased further in the combined treatment groups compared to the single Cd treatment groups (Figure 4d). These results suggest that, in terms of α-diversity, combined exposure to Cd and ENR led to an increase in the Chao index, ACE index, and Shannon index compared to the single exposure groups and a decrease in the Simpson index compared to the single exposure groups. This experiment investigated the differences in the composition of bacterial communities in zebrafish across five different treatment groups. PCA analysis (Figure 5a) revealed significant separation of bacterial communities between the L-Cd and L-CE groups, as well as H-Cd and H-CE groups (R^2^ = 0.6588, *p* = 0.002), where PC1 accounted for 39.41% of the total variance. Among them, PC1 mainly contained the OUT649, OTU696, OTU714, OTU741, OTU822, OTU657, and PC2 mainly included OUT687l, OTU751 and OTU663. PCoA analysis (Figure 5b) demonstrated significant separation of intestinal microbiota between single and combined exposure groups (PERMANOVA: R^2^ = 0.6692, *p* = 0.002), with the first two axes collectively explaining 88.94% of the total variability (PCo1: 60.84%; PCo2: 28.10%). This high cumulative variance suggests strong compositional differences among treatments.

Through the processing of intestinal samples, diversity data analysis was conducted for 15 samples, yielding a total of 263,591 optimized sequences with 55,328,742 base pairs (bp) and an average sequence length of 1049 bp. The species annotation results are summarized as follows: 1 domain, 1 kingdom, 14 phyla, 28 classes, 79 orders, 123 families, 258 genera, 407 species, and 541 Operational Taxonomic Units (OTUs). The top five species at the phylum level include *Pseudomonadota* (formerly known as *Proteobacteria*), *Fusobacteriota*, *Bacillota* (formerly known as *Firmicutes), Actinomycetota* (formerly known as *Actinobacteriota*), and *Bacteroidota* (formerly known as *Bacteroidota*), while the top five species at the genus level comprise *Aeromonas*, *Cetobacterium*, *ZOR0006*, *Shewanella*, and *Mycoplasma*.

**Figure 6:**
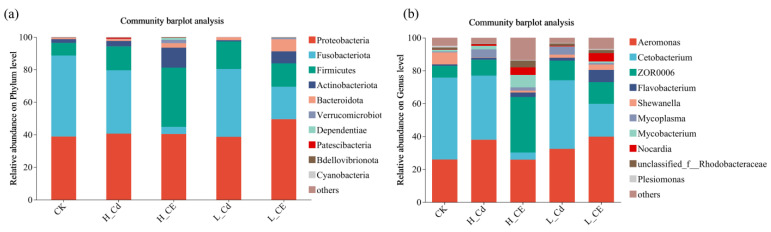
Composition analysis of intestinal flora of zebrafish. (**a**) Species composition map based on gate level; (**b**) Species composition map based on genus level.

At the phylum level among the five treatment groups (Figure 6a), the dominant bacterial phyla in the intestinal microbiota of zebrafish are *Pseudomonadota*, *Fusobacteriota*, *Bacillota*, and *Actinomycetota*. *Fusobacteriota* is the most dominant phylum in both the CK and L-Cd groups, accounting for 49.80% and 41.72%, respectively. *Pseudomonadota* is the most dominant phylum in the H-Cd, L-CE, and H-CE groups, accounting for 40.64%, 49.53%, and 40.44%, respectively. Compared to the CK group, the proportion of *Fusobacteriota* decreased, and the proportion of *Bacillota* increased in the Cd-alone exposure groups. When comparing the L-Cd and L-CE groups, the proportions of *Pseudomonadota* and *Actinomycetota* increased, while the proportions of *Fusobacteriota* and *Bacillota* decreased. In contrast, when comparing the H-Cd and H-CE groups, the proportion of *Fusobacteriota* decreased significantly, while the proportions of *Bacillota* and *Actinomycetota* increased significantly. At the genus level (Figure 6b), the dominant bacterial genera in the intestinal microbiota of zebrafish mainly include *Aeromonas*, *Cetobacterium*, and *ZOR0006*. Compared to the CK group, the proportions of *Aeromonas* and *ZOR0006* increased, while the proportion of *Cetobacterium* decreased in the Cd-alone exposure groups. When comparing the L-Cd and L-CE groups, the proportions of *Aeromonas* and *ZOR0006* increased, while the proportion of *Cetobacterium* decreased. In contrast, when comparing the H-Cd and H-CE groups, the proportions of *Aeromonas* and *Cetobacterium* decreased significantly, while the proportion of *ZOR0006* increased significantly.

**Figure 7:**
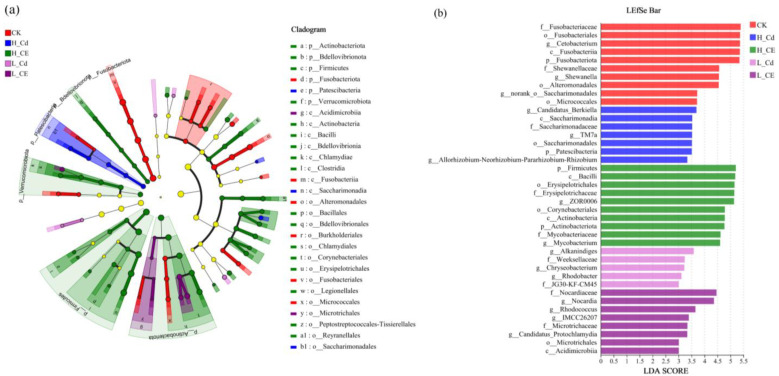
Significant difference analysis of dominant bacteria. (**a**) lefse multi-level species difference discrimination table; (**b**) LDA discrimination result table.

In this study, we conducted an in-depth analysis of bacterial community structure characteristics using LEfSe analysis. As shown in Figure 7, we screened and displayed indicator species with significant differences (LDA score > 3.0). The analytical results revealed distinct microbial community variations among the five treatment groups, with a total of 40 significantly different indicator species identified (*p* < 0.05). These included 10 characteristic species in the CK group, 5 in the L-Cd group, 7 in the H-Cd group, 8 in the L-CE group, and 10 in the H-CE group. The distribution patterns of these differential species reflect the specific effects of different treatment conditions on gut microbiota composition.

**Figure 8:**
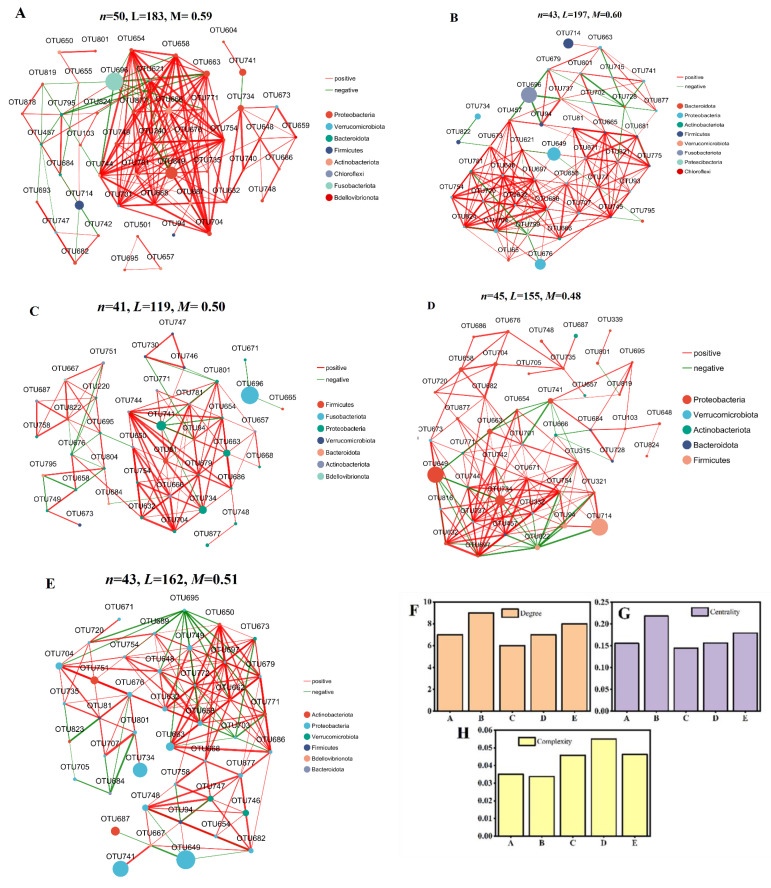
The co-occurrence network of bacterial of CK (**A**), L-Cd (**B**), L-CE (**C**), H-Cd (**D**) and H-CE (**E**) groups, and the related degree (**F**), centrality (**G**) and complexity (**H**). (Note: *n* represents the number of nodes, *L* indicates the number of edges, and *M* stands for modules).

Co-occurrence patterns of the gut microbiota were explored using network interaction analysis (Figure 8). In the CK group, the microbial interaction network consisted of 50 nodes and 183 edges (Figure 8A). In contrast, the number of nodes and edges decreased in the L-Cd (43 nodes and 197 edges) (Figure 8B), L-CE (41 nodes and 119 edges) (Figure 8C), H-Cd (45 nodes and 155 edges) (Figure 8D), and H-CE (43 nodes and 162 edges) (Figure 8E) groups, which indicated that the interaction between bacteria was gradually reduced after addition of high or low dose Cd and ENR. Among them, the close contact between microorganisms in CK, low-dose Cd, low-dose ENR, high-dose Cd and high-dose ENR groups was *Cerrucomicrobiota*, *Fusobacteriota*, *Fusobacteriota*, *Pseudomonadota* and *Pseudomonadota*, respectively. The “degree” of the microbial network is not only the quantitative parameter of topological structure but also the “fingerprint” of ecological function, stability and environmental response. The decline in the average degree may indicate ecological pressure. The average degree of microbial network decreased, indicating that the collaboration between microorganisms was weakened, and the community tended to be simplified to deal with the toxic environment. Additionally, the centrality of the microbial network is not only the quantitative parameter of topology but also the “baton” of ecological function, stability and environmental response. Under environmental disturbance, centrality distribution predicts the direction of network reconstruction. The centrality of the average betweenness of the network decreased, indicating that the connection between modules was weakened, and the community tended to be closed to resist toxicity. Except for the low-Cd group, there were no significant changes in the degree and centrality of network structures between the different treatment groups (Figure 8F,G). Microbial network complexity is not only a quantitative feature of topological structure but also a “control center” of ecosystem function, stability and host health. The improvement of network complexity was positively correlated with stability. Therefore, Cd and ENR were added in this study to improve the stability of the network (Figure 8H).

## 4. Discussion

### 4.1. Acute Toxicity of Cd and ENR to Zebrafish and Accumulation of Cd in Zebrafish Tissues Under ENR Stress

In real environments, the coexistence of multiple pollutants makes their interactions and environmental impacts more complex compared to single pollutants. Studying combined pollution and joint toxicity is crucial for accurately understanding the environmental stressors faced by organisms [45]. Aquatic organisms are typically exposed to a mixture of multiple pollutants, and even if individual pollutants are present at ineffective concentration levels, combined exposure may still pose greater ecological and health risks. For instance, the combined acute toxic effects of methoxyacrylate fungicides and succinate dehydrogenase inhibitors on tropical-clawed frog embryos exhibit synergism [46]. Luo et al. [47] found that the presence of microplastics enhances the accumulation of Cd in t *Bellamya aeruginosa* and exacerbates oxidative and DNA damage. The results of our experiments revealed a dose-effect relationship between the concentrations of Cd and/or enrofloxacin (ENR) and the mortality rate of zebrafish, with mortality increasing as concentrations rose. Acute toxicity tests showed that Cd was 2.13 times more toxic to zebrafish than ENR; the toxicity of combined exposure was 1.92 times that of Cd alone and 1.91 times that of ENR alone. Cd was more toxic to zebrafish than ENR, indicating that zebrafish are more sensitive to the toxic effects of the heavy metal Cd. Cd and ENR differ in their mechanisms of action and toxicity within zebrafish [25,48,49,50]. The toxicity of ENR is primarily manifested as organ damage and developmental toxicity under chronic exposure. Mechanistically, ENR induces mitochondrial fragmentation by promoting dephosphorylation of dynamin-related protein 1 (DRP1), which subsequently disrupts fatty acid transport into mitochondria. This cascade ultimately leads to lipid accumulation and oxidative stress [51]. Whereas the toxicity of Cd tends to be more associated with neurotoxicity and internal environment disruption under acute exposure, exerting its effects through disruption of calcium ion channels, excessive induction of ROS, and interference with metallothionein’s physiological functions [52]. Under combined stress from Cd and ENR, their toxic effects exhibit a trend of synergistic enhancement. This synergistic interaction likely stems from crosstalk between their respective molecular pathways, wherein ENR-aggravated oxidative stress and Cd-induced ROS generation form a positive feedback loop, ultimately exacerbating cellular apoptosis and organ dysfunction.

Cd exhibited distinct tissue-specific accumulation patterns in zebrafish. Experimental data revealed that after 20 days of exposure, the cadmium accumulation level in intestinal tissue was significantly higher than in other tissues, with concentrations being 9.54–31.60 times and 16.93–38.49 times those in liver and muscle tissues, respectively. Meanwhile, the cadmium accumulation in liver tissue was also significantly higher than in muscle tissue (1.22–1.77 times). These findings are consistent with previous studies, indicating that dietary intake is the primary route of Cd accumulation in zebrafish, and the liver, due to its detoxification, metabolic, and immune defense functions, serves as a critical organ for Cd accumulation [53]. Alvarado et al. [32] found that the Cd accumulation capacity in carp tissues followed the order of liver > gills > muscle. Similarly, Isan et al. [54] observed that after 11 days of exposure, the highest accumulation of Cd occurred in the liver of gilthead seabream, while lower levels were detected in the muscle and blood.

Furthermore, the metabolic detoxification and pollutant accumulation processes in organisms may be affected by interactions between pollutants. In this study, the Cd content in the co-exposure group was higher than that in the single-exposure groups, which may be attributed to the formation of Cd-ENR complexes. Studies have shown that antibiotics and heavy metals can easily form complexes when coexisting, thereby enhancing the absorption of heavy metals by organisms [55]. Therefore, the increased absorption of Cd in zebrafish under co-exposure conditions may be due to the uptake of both uncomplexed metals and Cd-ENR complexes. Similarly, Li et al. [38] found that the presence of ENR at low environmental concentrations significantly increased Cd accumulation in *Eisenia fetida*. Li et al. [56] also reported that co-exposure to polymyxin B (PMB) and arsenic (As) significantly enhanced the bioaccumulation of As in the tissues of *Metaphire guillelmi*. These findings from previous studies align with the conclusions drawn in this experiment.

### 4.2. Effects of Cd on Hepatic Antioxidant Enzymes and Oxidative Damage Markers Under ENR Exposure

Given that ENR promotes the bioaccumulation of Cd in zebrafish, this experiment further investigated the toxicological effects of co-exposure to ENR and Cd. Pollutants entering organisms can induce the production of reactive oxygen species (ROS) [57]. The antioxidant system can dynamically scavenge excess ROS in organisms; however, when the production rate of ROS exceeds the scavenging rate, it can induce oxidative stress [58,59], leading to damage to biomolecules such as lipids, proteins, and nucleic acids [25,60,61,62], thereby affecting cellular functions and physiological activities [63,64,65,66]. As the primary organ for toxicant metabolism, the liver is highly susceptible to pollutant-induced structural and functional damage [67]. The experimental results presented in this paper indicate that, whether exposed to Cd alone or in combination with ENR, the activities of SOD, CAT, and GSH-PX enzymes in the liver of zebrafish were significantly elevated during the initial exposure period, indicating that the antioxidant defense system was activated to protect zebrafish from oxidative stress. However, as exposure time increased, the activities of these three enzymes decreased, suggesting that the accumulation of Cd and/or ENR in the liver exceeded the adaptive capacity of the organism, leading to structural damage to tissues and organs and consequently reduced enzyme activities. In their study on the effects of heavy metal exposure on zebrafish, Chen et al. [68] found that under zinc, chromium, and mercury stress, the activities of SOD and CAT enzymes in zebrafish significantly increased during the early stages but almost diminished as the exposure duration extended. Chen et al. [69] observed that in mussels exposed to mercury and/or selenium, the activities of SOD and GSH-PX enzymes initially increased and then decreased over time. Excessive accumulation of malondialdehyde (MDA) can cause crosslinking and polymerization of life macromolecules such as nucleic acids and proteins, leading to changes in the structure and function of cell membranes. Therefore, MDA levels reflect the imbalance of the antioxidant system and the degree of damage to the body [70,71]. The experimental results presented here show that both single and combined exposures led to a significant increase in MDA content in zebrafish liver over time, indicating that oxidative stress damage and lipid peroxidation in the liver were aggravated with prolonged exposure. This result is consistent with the findings of Wang et al. [72] and Souid et al. [73], who reported a continuous increase in MDA content in the livers of greenling and gilthead seabream under Cd exposure. Within the same exposure time, the activities of SOD, CAT, and GSH-PX enzymes and MDA content in the liver of zebrafish in the combined exposure group were significantly higher than those in the Cd-only exposure group. These experimental results suggest that the presence of ENR enhances the damage to the antioxidant enzyme system in the liver of zebrafish caused by Cd while simultaneously exacerbating the degree of membrane lipid peroxidation. Chen et al. [74] found that co-exposure to Cd and nanoparticles (NPs) significantly increased the activities of SOD and CAT, as well as the MDA content in largemouth bass (*Micropterus salmoides*). Similarly, Liu et al. [75] observed that co-exposure to cadmium and prometryn exacerbated antioxidant damage in earthworms.

### 4.3. Effect of Cd on Intestinal Flora Under ENR

The gut microbiota plays a crucial role in the health and disease of the host, earning it the moniker of the host’s second genome [76]. As biomarkers of fish health and metabolic capacity, the gut microbiota influences fish physiology, development, and immunity, forming a barrier against pathogens in fish [77,78]. The functionality of the gut microbiota depends on its composition and structure, and when stimulated by exogenous substances, it may lead to gut microbiota disruption, causing inflammation or even death in the host [79]. To further investigate the toxic effects of Cd and/or ENR exposure on zebrafish, changes in their gut microbiota were analyzed. α-Diversity analysis showed that compared to the single Cd treatment group, the increase in Shannon index and the decrease in Simpson index in the combined treatment group indicated an increase in gut microbial community diversity. The Shannon index reflects the diversity of the gut microbiota, while the Simpson index indicates its evenness [80]. An increase in gut microbial diversity implies a decrease in the abundance of the original dominant microbiota and an increase in harmful microbiota [80]. Similar to our study, Liao et al. [81] found that after ingesting PSNP, largemouth bass exhibited an increase in the Shannon index and a decrease in the Simpson index, leading to disruption of gut microbial balance.

After exposure to Cd and/or ENR, changes occurred in the composition of the gut microbiota of zebrafish at different taxonomic levels. At the phylum level, the abundance of *Bacillota* and *Pseudomonadota* (except in the L-Cd group) increased, while the abundance of *Fusobacteriota* decreased. Compared to single exposure groups, the combined group showed an increase in *Actinomycetota* abundance and a decrease in *Fusobacteriota* abundance. *Pseudomonadota* is the dominant gut microbiota in fish; an increase in its number can lead to gut ecological imbalance and unstable community structure [82]. An increase in *Bacillota* abundance is also associated with metabolic imbalance and ecological disruption in gut microbial communities [83]. *Pseudomonadota*, *Bacillota*, and *Actinomycetota* are all considered pathogenic bacteria harmful to fish growth [84]. In contrast, *Fusobacteriota* is a host-friendly gut microbiota that provides vitamins to the body and promotes gut metabolism, thereby maintaining the balance of the gut microbiota [85]. Similar studies found that exposure of zebrafish to Cd^2+^ and/or NO_3_^−·^ led to a significant decrease in *Pseudomonadota* abundance and a significant increase in *Bacillota* abundance in the gut, resulting in gut microbiota imbalance and inducing gut inflammation, with the toxic effects of combined exposure being more severe [86]. At the genus level, dominant bacteria in the gut microbial community of zebrafish include *Aeromonas*, *Cetobacterium*, and *ZOR0006*. Compared to the single Cd exposure group, the relative abundance of *Cetobacterium* significantly decreased, while the relative abundance of *ZOR0006* significantly increased in the gut microbiota of zebrafish treated with the combined group. *Cetobacterium* is a dominant gut bacterium in fish and an important part of the microbial barrier function in fish [87]. The above experiments indicate that exposure of zebrafish to Cd and/or ENR reduces the abundance of probiotics in their gut and increases the abundance of pathogenic bacteria, leading to gut microbiota ecological imbalance. Moreover, compared to single exposure, combined exposure has a more severe toxic effect on the gut. Given that the gut microbiota participates in regulating various metabolic pathways of the host through intricate interaction networks, network analysis is crucial for comprehensively understanding and assessing the response of the gut microbiota to environmental changes [88,89]. In this study, the number of nodes and edges decreased in the L-CE (41 and 119), H-Cd (45 and 155), and H-CE (43 and 162) groups, while the number of gut microbiota edges increased in the L-Cd (43 and 197) group, indicating that exposure to Cd and/or ENR led to significant alterations in the network structure of the intestinal microbial community. Wei et al. [90] demonstrated that combined exposure to MPs and Cd-induced significant intestinal microbiota dysbiosis in *Cyprinus carpio*, characterized by increased relative abundances of *Pseudomonadota* and *Bacteroidota* phyla, along with elevated proportions of opportunistic pathogens, including *Pseudomonas* and *Pseudoxanthomonas* genera, potentially triggering intestinal inflammatory responses. Parallel studies revealed that exposure to Cu and MPs + Cu similarly disrupted the diversity and composition of gut microbiota in *Oreochromis niloticus*, particularly enhancing the prevalence of *Pseudomonadota* [91]. These findings collectively indicate that co-exposure scenarios exhibit synergistic toxicity, resulting in more severe disruption of intestinal microbial homeostasis. Such dysbiosis may not only facilitate the proliferation of antibiotic-resistant bacteria and compromise host immune defenses but also increase piscine susceptibility to pathogens, thereby posing potential ecological risks to higher trophic-level organisms through food chain transmission.

## 5. Conclusions

In this study, we systematically investigated the toxicological effects of cadmium Cd single exposure and Cd-ENR combined exposure using zebrafish as a model organism. Our results demonstrated that co-exposure to Cd and ENR produced additive toxic effects in zebrafish, significantly increasing ecological risks. The key findings include: (1) ENR enhanced Cd accumulation in intestinal, hepatic and muscular tissues; (2) Combined exposure induced more severe oxidative stress damage, which was characterized by an initial increase followed by a subsequent decrease in the activities of SOD, CAT, and GSH-Px, concomitant with sustained elevation of MDA levels; (3) The co-exposure also led to more pronounced gut microbiota dysbiosis, marked by reduction of beneficial bacteria and increase of pathogenic species.

These findings provide important scientific evidence for assessing heavy metal-antibiotic combined pollution in aquatic environments and highlight the necessity of comprehensive risk assessment. Future studies should further elucidate the molecular mechanisms underlying Cd-ENR interactions and develop corresponding pollution control strategies.

## Data Availability

The original contributions presented in this study are included in the article. Further inquiries can be directed to the corresponding author.

## References

[1] Zuccato E., Castiglioni S., Bagnati R., Melis M., Fanelli R. (2010). Source, occurrence and fate of antibiotics in the Italian aquatic environment. J. Hazard. Mater..

[2] Lu X., Li Y., Thunders M., Matthew C., Wang X., Ai X., Zhou X., Qiu J. (2018). Effect of enrofloxacin on the proteome of earthworms. Sci. Total. Environ..

[3] Carvalho I.T., Santos L. (2016). Antibiotics in the aquatic environments: A review of the European scenario. Environ. Int..

[4] Wu X., Zou H., Zhu R., Wang J. (2016). Analysis of Antibiotic Pollution Characteristics and Ecological Risk Assessment in the Waters of Gonghu Bay, Taihu Lake. Environ. Sci..

[5] Wang Q.J., Mo C.H., Li Y.W., Gao P., Tai Y.P., Zhang Y., Ruan Z.L., Xu J.W. (2010). Determination of four fluoroquinolone antibiotics in tap water in Guangzhou and Macao. Environ. Pollut..

[6] Teglia C.M., Perez F.A., Michlig N., Repetti M.R., Goicoechea H.C., Culzoni M.J. (2019). Occurrence, Distribution, and Ecological Risk of Fluoroquinolones in Rivers and Wastewaters. Environ. Toxicol. Chem..

[7] Ohore O.E., Qin Z., Sanganyado E., Wang Y., Jiao X., Liu W., Wang Z. (2022). Ecological impact of antibiotics on bioremediation performance of constructed wetlands: Microbial and plant dynamics, and potential antibiotic resistance genes hotspots. J. Hazard. Mater..

[8] Liu C., Yan H., Sun Y., Chen B. (2021). Contribution of enrofloxacin and Cu^2+^ to the antibiotic resistance of bacterial community in a river biofilm. Environ. Pollut..

[9] Qiu W., Liu T., Liu X., Chen H., Luo S., Chen Q., Magnuson J.T., Zheng C., Xu E.G., Schlenk D. (2022). Enrofloxacin Induces Intestinal Microbiota-Mediated Immunosuppression in Zebrafish. Environ. Sci. Technol..

[10] Bona M.D., Lizzi F., Borgato A., De Liguoro M. (2016). Increasing toxicity of enrofloxacin over four generations of *Daphnia magna*. Ecotoxicol. Environ. Saf..

[11] Jin X., Liu S., Zhang Z., Liu T., Li N., Liang Y., Zheng J., Peng N. (2023). Enrofloxacin-induced transfer of multiple-antibiotic resistance genes and emergence of novel resistant bacteria in red swamp crayfish guts and pond sediments. J. Hazard. Mater..

[12] Curpan A., Impellitteri F., Plavan G., Ciobica A., Faggio C. (2022). Review: *Mytilus galloprovincialis*: An essential, low-cost model organism for the impact of xenobiotics on oxidative stress and public health. Comp. Biochem. Physiol. Part C Toxicol. Pharmacol..

[13] Jyoti D., Sinha R., Faggio C. (2022). Advances in biological methods for the sequestration of heavy metals from water bodies: A review. Environ. Toxicol. Pharmacol..

[14] Tóth T., Zsiros O., Kis M., Garab G., Kovács L. (2012). Cadmium exerts its toxic effects on photosynthesis via a cascade mechanism in the cyanobacterium, Synechocystis PCC 6803. Plant Cell Environ..

[15] Liu G., Tong Q., He C., Fan R. (2004). Investigation and Research on Soil Cadmium Pollution. Sichuan Environ..

[16] Zhang M., Chen G., Luo Z., Sun X., Xu J. (2020). Spatial distribution, source identification, and risk assessment of heavy metals in seawater and sediments from Meishan Bay, Zhejiang coast, China. Mar. Pollut. Bull..

[17] El-Sorogy A., Al-Kahtany K., Youssef M., Al-Kahtany F., Al-Malky M. (2018). Distribution and metal contamination in the coastal sediments of Dammam Al-Jubail area, Arabian Gulf, Saudi Arabia. Mar. Pollut. Bull..

[18] (2002). Environmental Quality Standards for Surface Water.

[19] Zhu B., Shi Z., Wang X., Xu W., Yang W., Liao C. (2018). Spatial Distribution Characteristics and Source Identification of Heavy Metals in the Water Body of Anning River. Sichuan Metall..

[20] Lee S., Kim C., Liu X., Lee S., Kho Y., Kim W., Kim P., Choi K. (2021). Ecological Risk Assessment of Amoxicillin, Enrofloxacin, and Neomycin: Are Their Current Levels in the Freshwater Environment Safe?. Toxics.

[21] Mo L., Yang Y., Zhao D., Qin L., Yuan B., Liang N. (2022). Time-Dependent Toxicity and Health Effects Mechanism of Cadmium to Three Green Algae. Int. J. Environ. Res. Public Health.

[22] Ros M.T.L., Al-Enezi E., Cesarini E., Canonico B., Bucci C., Martins M.V.A., Papa S., Frontalini F. (2020). Assessing the Cadmium Effects on the Benthic Foraminifer Ammonia cf. parkinsoniana: An Acute Toxicity Test. Water.

[23] Gao D., Xu Z., Zhang X., Zhu C., Wang Y., Min W. (2013). Cadmium triggers kidney cell apoptosis of purse red common carp (*Cyprinus carpio*) without caspase-8 activation. Dev. Comp. Immunol..

[24] Naikoo M.I., Raghib F., Dar M.I., Khan F.A., Hessini K., Ahmad P. (2021). Uptake, accumulation and elimination of cadmium in a soil-Faba bean (*Vicia faba*)-Aphid (*Aphis fabae*)-Ladybird (*Coccinella transversalis*) food chain. Chemosphere.

[25] Sehonova P., Tokanova N., Hodkovicova N., Kocour Kroupova H., Tumova J., Blahova J., Marsalek P., Plhalova L., Doubkova V., Dobsikova R. (2019). Oxidative stress induced by fluoroquinolone enrofloxacin in zebrafish (*Danio rerio*) can be ameliorated after a prolonged exposure. Environ. Toxicol. Pharmacol..

[26] Raeeszadeh M., Khoei A.J., Parhizkar S., Rad F.T., Salimi B. (2023). Assessment of Some Heavy Metals and Their Relationship with Oxidative Stress and Immunological Parameters in Aquatic Animal Species. Biol. Trace Elem. Res..

[27] Wang N., Guo Z., Zhang Y., Zhang P., Liu J., Cheng Y., Zhang L., Li Y. (2020). Effect on intestinal microbiota, bioaccumulation, and oxidative stress of Carassius auratus gibelio under waterborne cadmium exposure. Fish. Physiol. Biochem..

[28] Yeşilbudak B., Erdem C. (2014). Cadmium Accumulation in Gill, Liver, Kidney and Muscle Tissues of Common Carp, *Cyprinus carpio*, and Nile Tilapia, *Oreochromis niloticus*. Bull. Environ. Contam. Toxicol..

[29] Chen H., Liu S., Xu X., Diao Z., Sun K., Hao Q., Liu S., Ying G. (2018). Tissue distribution, bioaccumulation characteristics and health risk of antibiotics in cultured fish from a typical aquaculture area. J. Hazard. Mater..

[30] Bhattacharya P., Mukherjee S., Mandal S.M. (2020). Fluoroquinolone antibiotics show genotoxic effect through DNA-binding and oxidative damage. Spectrochim. Acta Part A Mol. Biomol. Spectrosc..

[31] Liu X., Wang Z., Jin L., Huang J., Pu D., Wang D., Zhang Y. (2017). Effects of subchronic exposure to waterborne cadmium on H-P-I axis hormones and related genes in rare minnows (*Gobiocypris rarus*). Comp. Biochem. Physiol. Part C Toxicol. Pharmacol..

[32] Alvarado C., Ramirez J.M., Herrera-Lopez E.J., Cortez-Valladolid D., Ramirez G. (2020). Bioaccumulation of Metals in Cultured Carp (*Cyprinus carpio*) from Lake Chapala, Mexico. Biol. Trace Elem. Res..

[33] Chen M., Zhao H., Wang Y., Bekele T.G., Liu W., Chen J. (2019). Uptake and depuration of eight fluoroquinolones (FQs) in common carp (*Cyprinus carpio*). Ecotoxicol. Environ. Saf..

[34] Chen X., Guo J., Huang Y., Li Z., Yuan W., Zeng S., Zhu H., Zhong Y., Lin W., Lu H. (2023). Toxicity of o-phenylphenol on craniofacial cartilage development through ROS-induced oxidative stress in zebrafish embryos. Sci. Total Environ..

[35] Wang L., Zhang W., Wang J., Zhu L., Wang J., Yan S., Ahmad Z. (2019). Toxicity of enrofloxacin and cadmium alone and in combination to enzymatic activities and microbial community structure in soil. Environ. Geochem. Health.

[36] Gao M., Lv M., Han M., Song W., Wang D. (2016). Avoidance behavior of *Eisenia fetida* in oxytetracycline- and heavy metal-contaminated soils. Environ. Toxicol. Pharmacol..

[37] Lu X., Gao Y., Luo J., Yan S., Wang T., Liu L., Zhang Z. (2016). Interactive Effects of Tetracyclines and Copper on Plant Growth and Nutrient Uptake by *Eichhornia crassipes*. Clean-Soil Air Water.

[38] Li Y., Tang H., Hu Y., Wang X., Ai X., Tang L., Matthew C., Cavanagh J., Qiu J. (2016). Enrofloxacin at environmentally relevant concentrations enhances uptake and toxicity of cadmium in the earthworm *Eisenia fetida* in farm soils. J. Hazard. Mater..

[39] Zhao J., Liu Y., Pan B., Gao G., Liu Y., Liu S., Liang N., Zhou D., Vijver M.G., Peijnenburg W.J.G.M. (2017). Tannic acid promotes ion release of copper oxide nanoparticles: Impacts from solution pH change and complexation reactions. Water Res..

[40] Wang H., Wang Y., Wang Q., Lv M., Zhao X., Ji Y., Han X., Wang X., Chen L. (2022). The combined toxic effects of polyvinyl chloride microplastics and di(2-ethylhexyl) phthalate on the juvenile zebrafish (*Danio rerio*). J. Hazard. Mater..

[41] (2011). Chemicals-Fish Acute Toxicity Test.

[42] Belden J.B., Gilliom R.J., Lydy M.J. (2007). How Well Can We Predict the Toxicity of Pesticide Mixtures to Aquatic Life. Integr. Environ. Assess. Manag..

[43] Zhang Y., Liu S., Song X., Ge H. (2008). Prediction for the mixture toxicity of six organophosphorus pesticides to the luminescent bacterium Q67. Ecotoxicol. Environ. Saf..

[44] Mo L., Liang L., Qin L., Qin M., Gao H. (2018). Qualitative and Quantitative Assessment of the Toxicity Interactions of Mixtures of Four Heavy Metals and Two Pesticides on Vibrio fischeri. Asian J. Ecotoxicol..

[45] Vijver M.G., Peijnenburg W.J.G.M., De Snoo G.R. (2010). Toxicological Mixture Models are Based on Inadequate Assumptions. Environ. Sci. Technol..

[46] Wu S., Lei L., Liu M., Song Y., Lu S., Li D., Shi H., Raley-Susman K.M., He D. (2018). Single and mixture toxicity of strobilurin and SDHI fungicides to Xenopus tropicalis embryos. Ecotoxicol. Environ. Saf..

[47] Luo B., Li J., Wang M., Zhang X., Mi Y., Xiang J., Gong S., Zhou Y., Ma T. (2022). Chronic toxicity effects of sediment-associated polystyrene nanoplastics alone and in combination with cadmium on a keystone benthic species *Bellamya aeruginosa*. J. Hazard. Mater..

[48] Bautista C.J., Arango N., Plata C., Mitre-Aguilar I.B., Trujillo J., Ramírez V. (2024). Mechanism of cadmium-induced nephrotoxicity. Toxicology.

[49] Branca J.J., Fiorillo C., Carrino D., Paternostro F., Taddei N., Gulisano M., Pacini A., Becatti M. (2020). Cadmium-Induced Oxidative Stress: Focus on the Central Nervous System. Antioxidants.

[50] Du J., Liu Q. (2021). Enrofloxacin induces intestinal disorders of metabolome and microbiome in American shad (*Alosa sapidissima*). Aquac. Res..

[51] Wei X., Xu Y., Tan X., Lv W., Zhang D., He Y., Luo Z. (2023). Enrofloxacin (ENR) exposure induces lipotoxicity by promoting mitochondrial fragmentation via dephosphorylation of DRP1 at S627 site. Chemosphere.

[52] Liu Y., Chen Q., Li Y., Bi L., Jin L., Peng R. (2022). Toxic Effects of Cadmium on Fish. Toxics.

[53] Bo H., Feng J., Zhang X., Wang W., Li Q., Liu L., He C., Tian L. (2024). Research on Enrichment—Excretion Laws and Regulation of Strontium Ions in Zebrafish. Radiat. Prot..

[54] Isani G., Andreani G., Cocchioni F., Fedeli D., Carpene E., Falcioni G. (2009). Cadmium accumulation and biochemical responses in Sparus aurata following sub-lethal Cd exposure. Ecotoxicol. Environ. Saf..

[55] Hu X., Luo K., Ji K., Wang L., Chen W. (2022). ABC transporter *slr0982* affects response of *Synechocystis* sp. PCC 6803 to oxidative stress caused by methyl viologen. Res. Microbiol..

[56] Li L., Zhu D., Yi X., Su J., Duan G., Tang X., Zhu Y. (2021). Combined pollution of arsenic and Polymyxin B enhanced arsenic toxicity and enriched ARG abundance in soil and earthworm gut microbiotas. J. Environ. Sci..

[57] Birben E., Sahiner U.M., Sackesen C., Erzurum S., Kalayci O. (2012). Oxidative Stress and Antioxidant Defense. World Allergy Organ..

[58] Hajam M.E., Plavan G., Kandri N.I., Dumitru G., Nicoara M.N., Zerouale A., Faggio C. (2020). Evaluation of softwood and hardwood sawmill wastes impact on the common carp “*Cyprinus carpio*” and its aquatic environment: An oxidative stress study. Environ. Toxicol. Pharmacol..

[59] Mukherjee D., Saha S., Chukwuka A., Ghosh B., Dhara K., Saha N.C., Pal P., Faggio C. (2022). Antioxidant enzyme activity and pathophysiological responses in the freshwater walking catfish, Clarias batrachus Linn under sub-chronic and chronic exposures to the neonicotinoid, Thiamethoxam^®^. Sci. Total Environ..

[60] Muthulakshmi S., Maharajan K., Habibi H.R., Kadirvelu K., Venkataramana M. (2018). Zearalenone induced. embryo and neurotoxicity in zebrafish model (*Danio rerio*): Role of oxidative stress revealed by a multi biomarker study. Chemosphere.

[61] Plhalova L., Sehonova P., Blahova J., Doubkova V., Tichy F., Faggio C., Berankova P., Svobodova Z. (2020). Evaluation of Tramadol Hydrochloride Toxicity to Juvenile Zebrafish-Morphological, Antioxidant and Histological Responses. Appl. Sci..

[62] Sula E., Aliko V., Barcelo D., Faggio C. (2020). Combined effects of moderate hypoxia, pesticides and PCBs upon crucian carp fish, *Carassius carassius*, from a freshwater lake—in situ ecophysiological approach. Aquat. Toxicol..

[63] Barathinivas A., Ramya S., Neethirajan K., Jayakumararaj R., Pothiraj C., Balaji P., Faggio C. (2022). Ecotoxicological Effects of Pesticides on Hematological Parameters and Oxidative Enzymes in Freshwater Catfish, *Mystus keletius*. Sustainability.

[64] Freitas R., Silvestro S., Coppola F., Meucci V., Battaglia F., Intorre L., Soares A.M.V.M., Pretti C., Faggio C. (2019). Biochemical and physiological responses induced in *Mytilus galloprovincialis* after a chronic exposure to salicylic acid. Aquat. Toxicol..

[65] Pagano M., Savoca S., Impellitteri F., Albano M., Capillo G., Faggio C. (2022). Toxicological Evaluation of Acetylsalicylic Acid in Non-Target Organisms: Chronic Exposure on *Mytilus galloprovincialis* (Lamarck, 1819). Front. Physiol..

[66] Stara A., Pagano M., Capillo G., Fabrello J., Sandova M., Albano M., Zuskova E., Velisek J., Matozzo V., Faggio C. (2020). Acute effects of neonicotinoid insecticides on *Mytilus galloprovincialis*: A case study with the active compound thiacloprid and the commercial formulation calypso 480 SC. Ecotoxicol. Environ. Saf..

[67] Yan Z., Li S., Xing Q., Nan N., Qin G., Sang N. (2022). Research on the Differences in the Effects of PM2.5 from Different Cities on Liver Fibrosis in Mice. China Environ. Sci..

[68] Chen S., Zhou Q., Gao S., Liu X., Huang D., Yang H. (2019). Effects of Heavy Metal Ions of Zinc, Chromium and Mercury on Antioxidant Enzyme Activities in Zebrafish. Guangzhou Chem. Ind..

[69] Chen L., Zhang G., Chen J., Ren Z. (2011). Effects of Mercury and Selenium Exposure on the Antioxidant Enzyme System of Mytilus edulis. Asian J. Ecotoxicol..

[70] Nirwane A., Sridhar V., Majumdar A. (2016). Neurobehavioural Changes and Brain Oxidative Stress Induced by Acute Exposure to GSM900 Mobile Phone Radiations in Zebrafish (*Danio rerio*). Toxicol. Res..

[71] Chen M., Huang C., Pu D., Zheng Z., Yuan K., Jin X., Zhang Y., Jin L. (2015). Toxic Effects of CdSe/ZnS Quantum Dots on the Embryonic Development of Zebrafish. Environ. Sci..

[72] Wang X., Bian L., Hu Q., Qin B., Chang Q., Ying N., Wu Y., Yang L., Chen S. (2023). Effects of Cadmium on Acute Toxicity, Hepatic Antioxidant Capacity and Tissue Structure of Juvenile Thamnaconus modestus. Prog. Fish. Sci..

[73] Souid G., Souayed N., Yaktiti F., Maaroufi K. (2013). Effect of acute cadmium exposure on metal accumulation and oxidative stress biomarkers of *Sparus aurata*. Ecotoxicol. Environ. Saf..

[74] Chen M., Bao X., Yue Y., Yang K., Liu H., Yang Y., Yu H., Yu Y., Duan N. (2023). Combined effects of cadmium and nanoplastics on oxidative stress, histopatholog, and intestinal microbiota in largemouth bass (*Micropterus salmoides*). Aquaculture.

[75] Liu C., Zheng Y., Yang M., Sun S. (2025). Research on the Ecotoxicological Effects of the Combined Exposure of Cadmium and Prometryn on Earthworms. J. Southwest For. Univ. (Nat. Sci.).

[76] Backhed F., Manchester J.K., Semenkovich C.F., Gordon J.I. (2007). Mechanisms underlying the resistance to diet-induced obesity in germ-free mice. Proc. Natl. Acad. Sci. USA.

[77] Burns A.R., Stephens W.Z., Stagaman K., Wong S., Rawls J.F., Guillemin K., Bohannan B.J.M. (2016). Contribution of neutral processes to the assembly of gut microbial communities in the zebrafish over host development. ISME J..

[78] Clarke S.F., Murphy E.F., O’Sullivan O., Lucey A.J., Humphreys M., Hogan A., Hayes P., O’Reilly M., Jeffery I.B., Wood-Martin R. (2014). Exercise and associated dietary extremes impact on gut microbial diversity. Gut.

[79] Adak A., Khan M.R. (2019). An insight into gut microbiota and its functionalities. Cell. Mol. Life Sci..

[80] Xie S., Zhou A., Wei T., Li S., Yang B., Xu G., Zou J. (2021). Nanoplastics Induce More Serious Microbiota Dysbiosis and Inflammation in the Gut of Adult Zebrafish than Microplastics. Bull. Environ. Contam. Toxicol..

[81] Liao H., Liu S., Junaid M., Gao D., Ai W., Chen G., Wang J. (2022). Di-(2-ethylhexyl) phthalate exacerbated the toxicity of polystyrene nanoplastics through histological damage and intestinal microbiota dysbiosis in freshwater *Micropterus salmoides*. Water Res..

[82] Shin N., Whon T.W., Bae J. (2015). Proteobacteria: Microbial signature of dysbiosis in gut microbiota. Trends Biotechnol..

[83] Li Z., Yan L., Junaid M., Chen X., Liao H., Gao D., Wang Q., Zhang Y., Wang J. (2023). Impacts of polystyrene nanoplastics at the environmentally relevant and sub-lethal concentrations on the oxidative stress, immune responses, and gut microbiota to grass carp (*Ctenopharyngodon idella*). J. Hazard. Mater..

[84] Teame T., Wu X., Hao Q., Ding Q., Liu H., Ran C., Yang Y., Xia L., Wei S., Zhou Z. (2020). Dietary SWF^®^ enhanced growth performance and disease resistance in hybrid sturgeon (*Acipenser baerii x Acipenser schrenckii*) mediated by the gut microbiota. Aquac. Rep..

[85] Zheng Q., Cui L., Liao H., Junaid M., Li Z., Liu S., Gao D., Zheng Y., Lu S., Qiu J. (2023). Combined exposure to polystyrene nanoplastics and bisphenol A induces hepato- and intestinal-toxicity and disturbs gut microbiota in channel catfish (*Ictalurus punctatus*). Sci. Total Environ..

[86] Liu P., Wan Y., Zhang Z., Ji Q., Lian J., Yang C., Wang X., Qin B., Zhu L., Yu J. (2023). Toxic effects of combined exposure to cadmium and nitrate on intestinal morphology, immune response, and microbiota in juvenile Japanese flounder (*Paralichthys olivaceus*). Aquat. Toxicol..

[87] Sylvain F., Derome N. (2017). Vertically and horizontally transmitted microbial symbionts shape the gut microbiota ontogenesis of a skin-mucus feeding discus fish progeny. Sci. Rep..

[88] Lu J., Zhang X., Qiu Q., Chen J., Xiong J. (2020). Identifying Potential Polymicrobial Pathogens: Moving Beyond Differential Abundance to Driver Taxa. Microb. Ecol..

[89] De Vries F.T., Griffiths R.I., Bailey M., Craig H., Girlanda M., Gweon H.S., Hallin S., Kaisermann A., Keith A.M., Kretzschmar M. (2018). Soil bacterial networks are less stable under drought than fungal networks. Nat. Commun..

[90] Wei W., Yang Q., Xiang D., Chen X., Wen Z., Wang X., Xu X., Peng C., Yang L., Luo M. (2023). Combined impacts of microplastics and cadmium on the liver function, immune response, and intestinal microbiota of crucian carp (*Carassius carassius*). Ecotoxicol. Environ. Saf..

[91] Zhang F., Li D., Yang Y., Zhang H., Zhu J., Liu J., Bu X., Li E., Qin J., Yu N. (2022). Combined effects of polystyrene microplastics and copper on antioxidant capacity, immune response and intestinal microbiota of Nile tilapia (*Oreochromis niloticus*). Sci. Total Environ..

